# Reply: Balancing Promise and Evidence: Critical Perspectives on SGLT2 Inhibitors for Cardiotoxicity Prevention

**DOI:** 10.1016/j.jaccao.2025.01.016

**Published:** 2025-03-18

**Authors:** Ammar W. Bhatti, Bhargav Makwana, Sumanth Khadke, Sourbha S. Dani, Sarju Ganatra

**Affiliations:** Lahey Hospital and Medical Center, Burlington, Massachusetts, USA

We appreciate the thoughtful comments by Dr Karakasis and colleagues on our recent paper on the use of sodium-glucose cotransporter 2 inhibitors (SGLT2is) in the prevention of cancer therapy–related cardiac dysfunction (CTRCD) among individuals with type 2 diabetes.^1^

We agree with several points raised in the letter. We could not account for factors such as medication adherence, dosage, and therapy combinations, because of the limitations of the data. Recognizing several limitations, in addition to extensive propensity matching analysis, the higher E values found in our analysis indicated that a stronger unmeasured confounder would be required to nullify the observed association between the outcome and exposure.^1^

At a cellular level, the cardiotoxicity of anticancer agents culminates in cardiomyocyte death, leading to irreversible cardiac dysfunction.^2^ SGLT2is enhance cardiomyocyte survival via improved energy metabolism, mitochondrial biogenesis, autophagy, ketone body use, reduced endoplasmic reticulum stress, and ferroptosis.^2^ This may explain their observed protective effects across the spectrum of anticancer agents.

The cohorts were well balanced for the diagnosis of chronic kidney disease. After propensity score matching, 20.4% in the SGLT2i cohort and 21.1% in the no-SGLT2i cohort had chronic kidney disease at baseline. Moreover, the primary objective of our study was to evaluate the effect of SGLT2is on adverse cardiovascular events, as the safety outcomes have been extensively assessed previously.^3^ To effectively capture SGLT2i users, we used a retrospective database of patients with type 2 diabetes. Although our study was focused on this population, evidence from trials and real-world studies demonstrates the cardio-kidney-metabolic benefits of SGLT2i, including in patients without diabetes. On the basis of these findings and their cardioprotective mechanisms, we anticipate similar benefits in preventing CTRCD in patients without diabetes.^2^ Although social disparities in SGLT2i access are a concern, baseline ambulatory health care use, used as a surrogate for access, showed no cohort differences after propensity score matching.

The EMPACARD-PILOT (Empagliflozin in the Prevention of Cardiotoxicity) study reported reduced asymptomatic CTRCD incidence but was limited by a short follow-up period and smaller sample size.^4^ Although real-world studies remain hypothesis generating, ongoing trials such as PROTECT (Potential Protective Role of SGLT-2 Inhibitors for Chemotherapy-Induced Cardiotoxicity; NCT06341842), PROTECTAA (cardioPROTECTion with Dapagliflozin in Breast Cancer Patients Treated with AnthrAcycline - PROTECTAA TRIAL; NCT06304857), and EMPACT (Empagliflozin in the Prevention of Cardiotoxicity in Cancer Patients Undergoing Chemotherapy Based on Anthracyclines; NCT05271162) will offer crucial insights into SGLT2is’ potential for preventing and treating CTRCD across diverse populations and contexts.
